# The Linkage between Breast Cancer, Hypoxia, and Adipose Tissue

**DOI:** 10.3389/fonc.2017.00211

**Published:** 2017-09-25

**Authors:** Linda K. Rausch, Nikolaus C. Netzer, Josef Hoegel, Stephan Pramsohler

**Affiliations:** ^1^Hermann Buhl Institute for Hypoxia and Sleep Medicine Research, Bad Aibling, Germany; ^2^Department of Sports Science, University Innsbruck, Innsbruck, Austria; ^3^Division of Sports Medicine and Rehabilitation, Department of Medicine, University Ulm, Ulm, Germany; ^4^Institute of Human Genetics, University of Ulm, Ulm, Germany

**Keywords:** hypoxia, adipocytes, breast cancer, HIF-1α, HIF-2α

## Abstract

**Objective:**

The development of breast cancer cells is linked to hypoxia. The hypoxia-induced factor HIF-1α influences metastasis through neovascularization. Hypoxia seems to decrease the responsiveness to hormonal treatment due to loss of estrogen receptors (ERs). Obesity is discussed to increase hypoxia in adipocytes, which promotes a favorable environment for tumor cells in mammary fat tissue, whereas, tumor cells profit from good oxygen supply and are influenced by its deprivation as target regions within tumors show. This review gives an overview of the current state on research of hypoxia and breast cancer in human adipose tissue.

**Methods:**

A systematic literature search was conducted on PubMed (2000–2016) by applying hypoxia and/or adipocytes and breast cancer as keywords. Review articles were excluded as well as languages other than English or German. There was no restriction regarding the study design or type of breast cancer. A total of 35 papers were found. Eight studies were excluded due to missing at least two of the three keywords. One paper was removed due to Russian language, and one was dismissed due to lack of adherence. Seven papers were identified as reviews. After applying exclusion criteria, 18 articles were eligible for inclusion.

**Results:**

Two articles describe the impairment of mammary epithelial cell polarization through hypoxic preconditioning. A high amount of adipocytes enhances cancer progression due to the increased expression of HIF-1α which causes the loss of ER α protein as stated in four articles. Four articles analyzed that increased activation of HIF’s induces a series of transcriptions resulting in tumor angiogenesis. HIF inhibition, especially when combined with cytotoxic chemotherapy, holds strong potential for tumor suppression as stated in further four articles. In two articles there is evidence of a strong connection between hypoxia, oxidative stress and a poor prognosis for breast cancer via HIF regulated pathways. Acute hypoxia seems to normalize the microenvironment in breast cancer tissue and has proven to affect tumor growth positively as covered in two articles.

**Conclusion:**

This review indicates that the development of breast cancer is influenced by hypoxia. A high amount of adipocytes enhances cancer progression due to the increased expression of HIF-1α.

## Introduction

Breast cancer is the most commonly diagnosed cancer in women (1). In 2012, over a million new cases were identified and figures are rising due to late diagnosis at already quite advanced cancer stages (World Cancer Research Fund International, 2012) (2). Breast cancer represents 25% of all cancer types in women and is the fifth most common cause of death. It is classified into three main groups (3). The hormone receptor (HR) positive group, which expresses estrogen receptor (ER) or progesterone receptor (PR); the epidermal growth factor receptor 2 (HER2) positive group and the triple-negative breast cancer (TNBC) group without expression of ER, PR, and HER2. 90% of breast cancer patients die in consequence of metastasis most commonly found in bone tissue (4).

Several prospective, epidemiological studies show that there is a direct relationship between obesity and cancer (5–9). Especially, the manifestation of breast cancer seems to be linked to obesity (10). Notably, female obese breast cancer patients show a less sufficient response to the same dosage of chemotherapy compared to female lean breast cancer patients (11). In premenopausal women, the risk for breast cancer is reduced with increasing body mass index (BMI). Thus, postmenopausal women are at higher risk for breast cancer development if BMI is increased (10). There is a strong association between BMI and breast cancer in ER−/PR+ receptor positive breast cancer types as found in a dose–response meta-analysis (12). This could be due to an increase in sex-hormones triggered by an increase in estradiol production of adipose tissue, caused by a higher activity of aromatase enzymes (13). Adipose tissue is divided into brown adipose tissue (BAT) and white adipose tissue (WAT). BAT is only 50 g compared to kilograms of WAT, which is an endocrine organ producing a large number of adipokines and cytokines (14). In the presence of hypertrophy, the protein synthesis of white adipocytes is changed toward producing pro-inflammatory adipokines, such as tumor necrosis factor-alpha. On the contrary, adiponectin is an anti-inflammatory adipokine with cardio-protective and anti-tumor actions. Dysfunctional adipose tissue in obesity causes defective adipokines with increased levels of pro-inflammatory factors (14). The currently available therapies for advanced breast cancer stages in obese women seem to achieve a rather poor clinical outcome. Conclusively, a long-lasting reduced-calorie diet seems to lower the risk for breast cancer (15).

It remains difficult to identify single impact factors as dietary changes, energy balance, amount of physical activity, and obesity on cancer development and progression (16, 17). It also remains unclear if the higher amount of adipose tissue and the resulting tissue hypoxia in obesity contributes to the development of cancer. Especially, the elevated activation of HIF’s seems to increase metastasis and worsen the prognosis of patient survival (18). Intra-tumoral partial pressure of oxygen (PO_2_) is decreased by 20% compared to healthy tissue (19). PO_2_ values below 10 mmHg have shown to drive cancer growth, metastasis, and mortality. In cancer tissue, oxygen supply can be restrained due to the proliferation of vessels. Therefore, HIF’s, as the key factors of hypoxic cancer cells, seem to stimulate inflammation and angiogenesis (18).

The concurrence of adipose tissue hypoxia to cancer development is not fully explained, but tumors are most likely surrounded by adipose tissue (20–22). Hence, it is likely that such a malignant environment may promote tumor development (22).

## Methods

**Figure 1:**
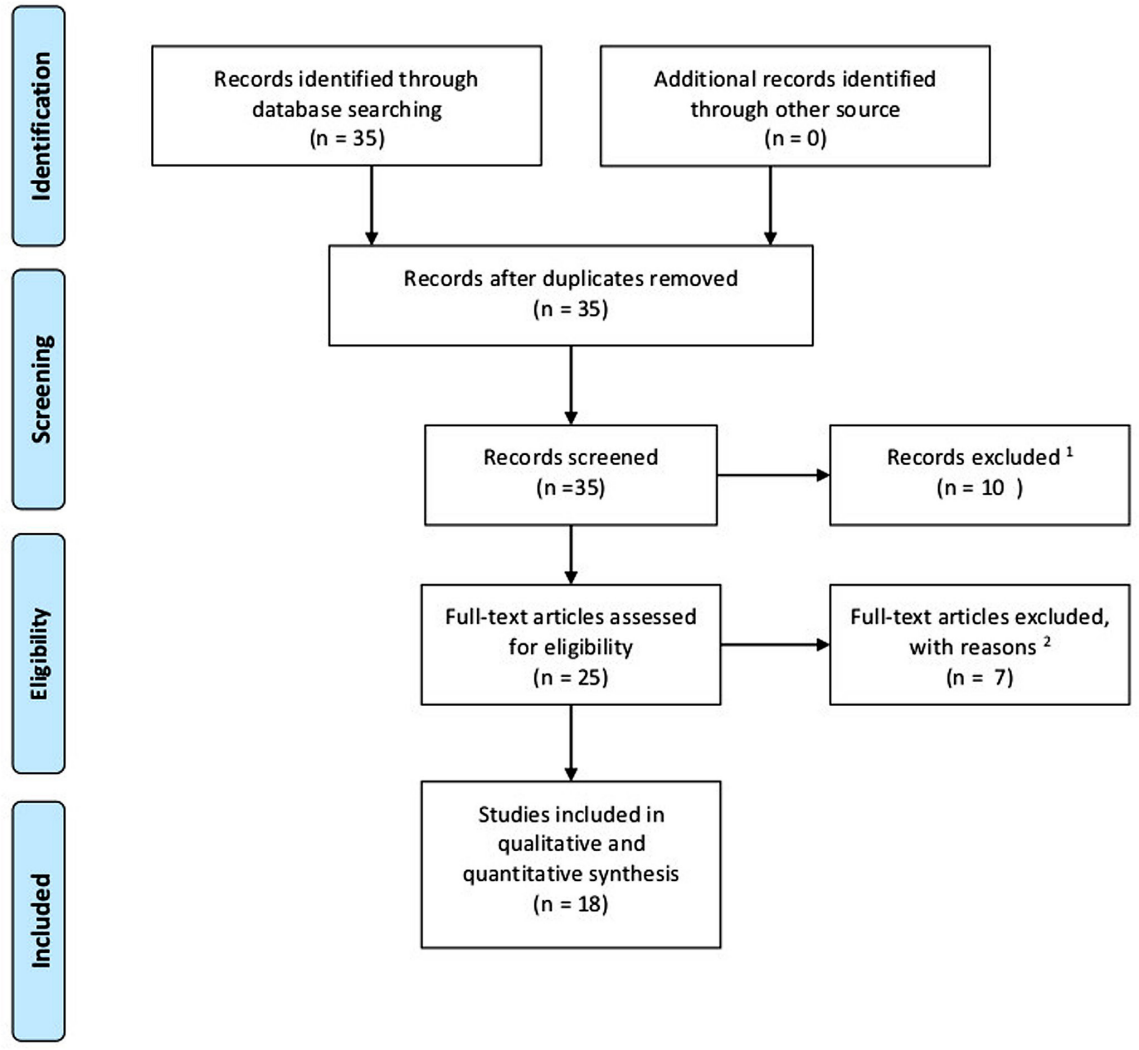
Flow diagram according to PRISMA-2015 protocols displaying process of literature identification, screening, eligibility, and inclusion. ^1^Language other than English or German; at least two of the three keywords missing. ^2^Fulltexts identified as reviews.

A literature search was conducted according to preferred reporting items for review and meta-analysis protocols (PRISMA-2015) statement. *Via* PubMed (2000–2016) search and manual searches of reference lists, studies examining the relationship between hypoxia, adipocytes, and breast cancer were identified. The keywords for the search were (*hypoxia* and/or *adipocyte*) and *breast cancer*. Articles had to be in English or German language. Review articles were excluded. There was no restriction regarding study design or certain breast cancer types. After this search, a total of 35 papers were identified. After title and abstract evaluation, eight studies were excluded due to lack of coherences with the topic. Out of four papers not offering open access, one paper was excluded due to Russian language, and another paper was also excluded due to lack of coherence. After assessing full-text articles for eligibility, seven papers were identified as reviews. Finally, 18 articles were eligible for inclusion in this review and selected for analysis. Figure 1 shows a flow diagram according to PRISMA-2015 protocols displaying the process of literature identification, screening, eligibility, and inclusion.

## Results

After the final evaluation of the 18 included articles, six on-topic categories were identified. Two studies identify the impact of hypoxic conditioning on malignant and non-malignant mammary epithelial cells. Two studies examine the role of hypoxic adipocytes in the development of breast cancer cells. Five studies approach the activation of HIF’s occurring in hypoxic adipocytes, which promotes breast cancer cell growth. Two studies identify the distinct biochemical responses of the body responsible for HIF inhibition. Three studies investigate medical interventions for HIF inhibition and limitation of breast cancer cell growth. Two studies express alternatives to drug cure of breast cancer inhibiting breast cancer *via* HIF pathways. Table 1 displays a study summary of HIF-related effects through different physiological and biochemical pathways on breast cancer progression.

**Table 1:** Study summary of HIF-related effects through different physiological and biochemical pathways on breast cancer progression.

	HIF activity	Physiological Effects	Effects on cancer progression
Denzel et al. (23)	↓	Reduced pulmonary metastasis	↓
Yao-Borengasser et al. (24)	↓	Reduction of ER gene expression	↓
Xiang et al. (25)	↓	Inhibition of HSP90	↓
Liapis et al. (26)	↓	Evofosfamide binds to hypoxic bone cell	↓
Samanta et al. (27)	↓	Paclitaxel or gemcitabine alternate HIF expression in triple-negative breast cancers (TNBCs)	↓
Hardman et al. (28)	↓	Dietary with omega three fatty acids	↓
Wang et al. (29)	↑	Increase of microvesicles	↑
Chaturvedi et al. (30)	↑	Increased signaling between BCCs and mesenchymal stem cells (MSCs)	↑
Gehmert et al. (31)	↑	Hypoxia and inflammation lead to migration of MSCs	↑
Seifert et al. (32)	↑	TCDD inhibits ERα signaling in MCF7 cells	↑
Luo et al. (33)	↑	Reprogramming of glucose metabolism	↑
Siclari et al. (34)	↑	Encoding adrenomedullin	↑
Vaapil et al. (35)	↑	Promoting metastasis	↑
Pahlman et al. (36)	↑	Failed lactation in mammary epithelium	↑
Krutilina et al. (37)	↑	Increase of micRNA	↑
Martinez-Outschoorn et al. (38)	↑	Endorsed autophagy	↑
Milane et al. (39)	↑	Increased glycolysis	↑
Jones et al. (40)	↑	Moderate-intensity exercise	↓

## Hypoxic Preconditioning

The most important element for tumor growth is the development of tumor vasculature (41–43). This vasculature is highly disorganized and constantly changing due to blood vessel gain and loss. A consequence of this alteration is the fluctuation of oxygen- and glucose levels, which result in heterogeneous states of hypoxia, anaerobic, and aerobic glycolysis (42). If a cell happens to be above its diffusion limit of oxygen, chronic hypoxia occurs (44). Transient hypoxia occurs due to local oxygen depletion (44). As a result of the fluctuant oxygenation within a tumor, it is possible that the hypoxia-induced glycolysis pre-conditions cancer cells for aerobic glycolysis (45). Increased glycolysis with and without the presence of oxygen is an important indicator for cancer and the connecting link between multidrug-resistant breast cancer cells and hypoxia (46–49). Milane et al. (39) extracted proteins of TNBC and ovarian cancer cell lines pre-exposed to either normoxic or hypoxic conditions. The TNBC cell line MDA-MB-231 experienced the most significant hypoxic transformation with an increase in all glycolytic proteins glucose transporters (GLUT-1 and GLUT-3), hexokinase 1 and 2, phosphofructokinase (PFK), aldolase, glyceraldehyde-3-phosphate dehydrogenase (GAPDH), phosphoglycerate kinase (PGK), enolase, pyruvate kinase, and lactate dehydrogenase (LDH). That indicates that each cell line has a time-specific threshold for hypoxic transformation inducing glycolysis (39). This finding is based on malignant breast cancer cells, but little is known about the effects of hypoxia on non-malignant cells. Vaapil et al. (35) cultivated normal human primary breast epithelial cells and non-malignant mammary epithelial MCF-10A cells under hypoxia and normoxia. The breast epithelial cells with high HIF-levels were found to be immature compared to the well-oxygenated cells. Due to the fact that constant cell proliferation is followed by high HIF-levels in certain compartments of the tumor, cellular differentiation of non-malignant human mammary epithelial cells is restrained (35).

Hypoxic preconditioning impairs polarization and organization of mammary epithelial cells and enhances cancer manifestation and progression.

## The Role of Hypoxic Adipocytes in the Development of Breast Cancer Cells

Obesity is accompanied with the development of hypoxic fat tissue and an increase of oxidative stress (50, 51). Conditioned by rising cell size, oxygen (O_2_) diffusion is decreased and vascular growth impaired in the hypoxic fat tissue (52). The mitochondrial production of excessive free fatty acids leads to increased procreation of reactive oxygen species (ROS), which causes oxidative stress (53, 54). As a consequence, the production of adipokines, cell signaling proteins secreted by adipose tissue, is defective and leads to angiogenesis and inflammation (50). This reaction chain creates a pro-malignancy setting in epithelial tissue for the development of breast cancer cells. Gehmert et al. (31) isolated mesenchymal stem cells (MSCs) from subcutaneous fat tissue. Breast cancer cells were injected into mammary fat pad and it showed that MSCs migrated primarily toward an inflammatory milieu in tumor stroma and vasculature independent of biological processes causing inflammation. It is suggested that the migration of MSCs depends on cancer-secreted cytokines due to the lack of inflammatory response by the immune system (31). Furthermore, Yao-Borengasser et al. (24) co-cultured the progressive breast cancer cell line MCF7 with human adipocytes. The MCF7 cell line is the most investigated cell line to analyze the cross talk of estrogen and ERα protein (estrogen receptor alpha protein) (32). They found a decreased level of ERα protein caused by deregulated adipocytes under hypoxic cell conditions. In human adipocyte cells, HIF-1α gene expression was increased and accompanied by a reduction of ER gene expression. With the loss of ERα protein, the tumor progresses and hormone therapy is less efficient (24). Seifert et al. also analyzed MCF7 cell lines cultivated under mild hypoxic conditions (5% of O_2_ for a duration of 6 h) (32). These cell lines were exposed to TCDD (2,3,7,8-tetrachlorodibenzo-para-dioxin), a pollutant causing a variety of biochemical and toxic effects, accumulating in adipose tissue. The prevalence of breast cancer cells was significantly higher due to the positive correlation with increased TCDD serum levels. TCDD reduces the hypoxia-induced stabilization and activation of HIF-1α (32).

Denzel et al. states that drug inhibition of the pro-angiogenic HIF-1α pathway only leads to temporary improvement and breast cancer resists treatment after a limited time frame (23). The effect of HIF on changes in human adipocytes inclines with extended exposure time (55, 56). They investigated cellular functions of adiponectin in breast cancer cells creating an adiponectin null mouse model of mammary cancer. The treatment of adiponectin leads to a reduction of human breast cancer cells due to adiponectins’ cancer-protective functions. Vessel density is restrained through tumor vasculature because of adiponectin deficit. This limits the supply of oxygen and nutrients (23). Therefore, high adiponectin levels in women are associated with a lower risk of breast cancer and tumor metastasis (57, 58).

A high amount of adipocytes enhances cancer progression due to the increased expression of HIF-1α which causes the loss of ERα protein. Thus, a high amount of the peptide hormone adiponectin appears to be cancer protective.

## Activation of HIF’s and the Impact on Breast Cancer Cell Growth

HIF-1α and HIF-2α are linked to breast cancer metastasis and poor patients’ survival (21, 59). The expression of HIF-1α and HIF-2α occurs differently during separate phases of mammary gland development and function (36). Selective inhibition of HIF-1α expression in mammary epithelium leads to lactation failure and in breast cancer models to increased tumor growth (60–62). Pahlman et al. investigated the separate phases using different mouse models with MCF-7 breast cancer cells. They found that the regulation and expression of the two factors and its subunits is not merely dependent on the availability of oxygen (36). Under hypoxic condition, HIFs are stabilized. In a malignant setting, the activation of HIF-induced transcriptions is implemented in extracellular proteolytic activity, invasion, and angiogenesis (36). Wang et al. cultivated TNBC cell lines that were exposed to hypoxia (29). These cells increased their production of microvesicles due to HIF expression. Microvesicles contain proteins that stimulate the invasion and metastasis of breast cancer cells (63). Chaturvedi et al. found that tumor growth, which promotes signals between TBNC’s and MSCs is stimulated by HIF activity (30). HIF activates transcription genes, which encode proteins that play a role in proliferation of breast cancer cells. As stated by Luo et al., some of these proteins only interact with HIF-1α, but not with HIF-2α. The consequence is the reprogramming of the glucose metabolism of breast cancer cells which generates macromolecular blocks, such as amino acids and acetyl CoA, that release more breast cancer cells (33). Furthermore, Siclari et al. (34) identify adrenomedullin as a 52-amino acid peptide for which gene transcription is increased by the HIF-1α pathway. This peptide stimulates angiogenesis and proliferation. Many cancer types release adrenomedullin and its receptors which is indirectly connected to poor survival probability (64).

Taken together, increased activation of HIF’s induces a series of transcriptions resulting in tumor invasion and angiogenesis. Adrenomedullin is one of them which plays a major role.

## Inhibition of HIF’s and the Impact on Breast Cancer Cell Growth

Tumor hypoxia contributes to a great degree to treatment failure and increased patients’ mortality for a broad range of malignancies (65). Hypoxic regions within a solid tumor contain cancer cells that resist conventional chemotherapy or radiotherapy (66). This leads to cancer recurrence and metastasis (67). HIF’s activate two main transcription processes. First, the gene expression of vascular endothelial growth factors (VEGFs) which contributes to vascularization (68, 69) and, second, the expression of proteins regulating the change from mainly oxidative to glycolytic metabolism (70). The identification of chemical HIF inhibitors and their mechanisms has been a relevant target in anti-cancer research (71). The difficulty in analyzing the development of HIF inhibitors is the lack of specificity. In the ER−/PR+ cancer group, there are already appropriate receptor-blocking inhibitors in use while we still lack comparable methods for TNBCs (72). This type of cancer is associated with increased mortality compared to other types. Inhibition of HIF’s and its target genes in consequence could provide a feasible method for tumor suppression.

Xiang et al. showed that in human breast cancer cell cultures the drug Ganetesip inhibits, among others, the expression of the heat shock protein 90 (HSP90). The lack of HSP90 leads to a degeneration of HIF-1α (25). The distribution of HIF-1α was decreased by 35% in breast cancer cells and the expression of VEGFs was reduced as well. The inhibition of HSP90 resulted in a reduction of tumor weight and -growth (25). Another prodrug exhibiting hypoxia-selective cytotoxicity on breast cancer cells is Evofosfamide (TH-302). Liapis et al. show that by binding to hypoxic bone cells, the drug is able to destroy 50–90% of hypoxic cancer cells in bone tissue (26). The advantage of Evofosfamide therapy seems to be greatest when combined with cytotoxic chemotherapy. The combination of chemotherapy and HIF inhibiting drugs is also suggested by Samanta et al. This is based on the finding that paclitaxel as well as gemcitabine change the activity of HIF expression and transcription in human TNBC cell lines (27).

HIF inhibition, especially when combined with cytotoxic chemotherapy, holds strong potential for tumor suppression as well as for the reduction of metastasis.

## Self-Regulating Mechanisms of the Human Body and Its Impact on HIF Expression

The human body offers several regulating mechanisms affecting HIF expression and in consequence tumor progression. One important regulating mechanism seems to be the distinct expression profiles of microRNAs that are associated with molecular subgroups and pathological characteristics in breast cancer (73). Krutilina et al. (37) link the expression of microRNAs to a HIF-1α dependent hypoxic response. A growing number of microRNAs have been described as oncogenes and tumor suppressors. Within solid tumors, microRNAs have proven to be downregulated which causes a higher expression of HIF-1α. In consequence, the downregulation of microRNAs withholds a higher probability for metastasis (74–76). One of the most frequently deregulated microRNAs-encoding genes in human cancer is the polycistronic MIR17HG gene, which encodes six microRNAs including miR-18a. Increased expression of miR-18a in MDA-MB-231 breast cancer cell lines has shown to reduce primary tumor growth and lung metastasis and miR-18a inhibition promotes tumor growth and lung metastasis (37). Besides other self-regulating mechanisms of the human body, the regulation of autophagy heavily affects the development and growth of breast cancer. Autophagy is a catabolic process responsible for the systematic degradation and recycling of cellular components (38). Yao-Borengasser et al. show that hypoxia and oxidative stress promote autophagy and support a pro-malignancy setting in epithelial tissue for the development of breast cancer cells (24, 77). Furthermore, as stated by Martinez-Outschoorn et al. (38) in some cases, autophagy promotes tumor progression while in other cases autophagy has shown to have tumor-suppressive effects. Increased HIF expression promotes autophagy and stromal caveolin-1 is degraded. Caveolin-1 appears to be tumor suppressive and low levels carry a poor prognosis for tumor development for the patient (78–80).

The human body offers a range of tumor affecting mechanisms that are not fully understood. Nevertheless, there seems to be a strong connection between hypoxia, oxidative stress, and a poor prognosis for breast cancer via HIF-regulated pathways.

## Alternatives to Drug Cure of Breast Cancer Inhibiting Breast Cancer Cell Growth

Physical activity is discussed as a supportive factor for breast cancer therapy and has proven to be quite effective (81, 82). Jones et al. (40) investigated effects of moderate aerobic exercise on tumor characteristics, such as vascularization, angiogenesis, and metabolism. MDA-MB-231 breast cancer cell line implanted mice were randomly assigned to voluntary wheel running. Moderate aerobic exercise has shown to increase intra tumor vascularization, which leads to normalization of tissue environment. This is one of the first studies to evaluate the impact of an exercise intervention on the microenvironment in cancer tissue (40). In contrast to other studies exercise-induced high concentration of HIF is associated with a normalization of cancer microenvironment. This is thought to improve oxygenation and removal of by-products in the long run. In several other studies, regular moderate-intensity exercise is associated with a 30–50% reduction in the risk of mortality in cancer, a fact which supports this finding (83, 84). Physical exercise as well as dietary interventions has shown to affect tumor growth and progression. Hardman et al. investigated the effect of an omega-3 fatty acids enriched diet on mice bearing MDA-MB-231 breast cancer cells. This dietary delays tumor growth and vascularization which could be ought to the reduction of oxygen radicals and HIF expression in consequence (28).

Acute hypoxia seems to normalize the microenvironment in breast cancer tissue and has proven to affect tumor growth and progression positively.

## Conclusion

There seems to be a strong linkage between adipose tissue hypoxia and the development, growth, and progression of breast cancer. HIF-1α and its target genes play a strong role in driving breast cancer cell proliferation. A high amount of adipocytes enhances cancer progression due to the increased expression of HIF-1α which causes the loss of ERα protein. Thus, a high amount of the peptide hormone adiponectin appears to be cancer protective. On the other hand, tissue hypoxia seems to provide a feasible pathway for the identification of cancer cells and their degeneration. Physical activity shows to improve tissue hypoxia and to reduce adipose tissue and is very likely to improve prognosis as well as therapy outcome in breast cancer (85).

## Author Contributions

LR and SP: literature search and analysis and writing manuscript; JH: writing manuscript; and NN: designing research strategy, optimization of keywords, and revising manuscript.

## Conflict of Interest Statement

We declare that the research was conducted in the absence of any commercial or financial relationships that could be construed as a potential conflict of interest.
